# Underserved and overburdened: an ethnographic examination of rural animal sheltering

**DOI:** 10.3389/fvets.2026.1793261

**Published:** 2026-03-09

**Authors:** Laura A. Bray, Lori L. Jervis

**Affiliations:** 1 Center for Applied Social Research, University of Oklahoma, Norman, OK, United States; 2 Department of Anthropology, University of Oklahoma, Norman, OK, Unites States

**Keywords:** companion animal welfare, qualitative research, rural communities, shelter animal relocation, sheltering

## Abstract

**Introduction:**

In the US, rural communities face unique challenges to companion animal sheltering and welfare. Smaller municipalities often serve socially vulnerable populations but lack the fiscal and administrative capacity to provide robust public services, leaving animal welfare under-resourced, and deprioritized relative to other community needs.

**Methods:**

This study draws on interviews and ethnographic observations with shelter workers and animal rescuers in Oklahoma, a state with a large rural population, to better understand the organization and challenges of rural animal welfare services.

**Results:**

Findings show large “shelter deserts” in rural areas, where many residents have limited or no access to public animal services and killing remains the predominant form of population control. As a result, the bulk of rural animal welfare work falls on volunteer or poorly paid rescuers. While some rural communities have successfully transitioned to “no kill” through relocation programs that move shelter animals out of state, animal welfare organizations continue to struggle with funding, staffing, and market access. Through three case studies, this article illuminates the common challenges and varied strategies rural communities employ to address service gaps and pursue the “no-kill” ideal in the absence of a sustained public commitment to providing “standard of care” animal welfare services.

**Discussion:**

We conclude by discussing future research that can assess the generalizability of our findings and implications for animal welfare policy.

## 1 Introduction

In the US, 46 million people live in rural areas (1), where they are more likely than urban residents to keep pets (2). Despite higher rates of pet ownership, rural residents often have less access to basic animal care (3). Greater levels of economic insecurity create additional hardships for rural pet owners (1), creating a strong need for rural animal welfare services to fill the gaps. Yet limited fiscal and administrative capacity means that rural and small-town municipalities often struggle to support robust public services (4, 5). As a result, rural animal welfare services differ significantly in size, scope, and quality from their urban counterparts.

Shelter data shows that rural places face significant challenges and, as a result, have worse outcomes for animals. Although rural shelters deal with fewer total animals than urban ones, they intake more than double the animals per capita (10.7 animals per 1,000 population in urban counties vs. 25 per 1,000 population in rural counties) (6). Tellingly, shelter intake in the *least* socially vulnerable rural areas (16.1 animals per 1,000 population) is nearly as high as the *most vulnerable* urban areas (17.4 animals per 1,000 population). Rural communities also struggle with increased risk of owner surrender (7) coupled with lower adoption rates (6), placing animals at greater risk of dying in rural shelters (8).^1^

Despite widely acknowledged geographic differences, nearly everything we know about animal sheltering and welfare appears to derive from large urban organizations. Ethnographic studies, for example, have analyzed the policies, operations, and culture of shelters in major cities like Los Angeles, California (9), Charlotte, North Carolina (10), and other large metropolitan areas [e.g., (11–13)]. Quantitative analyses of shelter outcomes also tend to focus on urban shelters with greater capacity to keep detailed organizational records (14–16). This urban bias has created a blind spot in our understanding of rural animal welfare services. To date, peer reviewed studies on rural animal sheltering and welfare remain virtually non-existent [exceptions to this pattern include (17, 18)].^2^

The history of sheltering may help explain the urban bias of existing studies. The US sheltering system first emerged and developed in the post-Civil War era as industrialization and urbanization brought together increasing numbers of people and animals in cities. Social anxieties and public health concerns over free-roaming dogs spurred the rise of “dog catchers” and city pounds to remove animals from the streets (19, 20). The earliest humane societies in New York City (1866), Boston (1868), and Philadelphia (1869)—initially modeled after the Royal Society for the Prevention of Cruelty to Animals in London (1824)—came about in response to the brutality of public animal culling and sought to both rehome animals and administer more humane deaths. The no-kill movement, beginning in the 1990s, likewise first found its foothold in large cities. In California, the San Francisco Society for the Prevention of Cruelty to Animals (SPCA), an early adopter of now common shelter practices like low-cost sterilization and creative marketing, declared San Francisco the first no-kill city in 1989. The urban-centered focus of animal welfare and the no-kill movement continues today. The vast majority of organizations that fund sheltering work and disseminate policy are headquartered in urban areas, with the notable exception of Best Friends Society for Animals in Kanab, Utah.

Although the history of rural sheltering has yet to be written, both animal welfare organizations and contemporary humane practices appear to have diffused more slowly outside of urban areas. Early no-kill organizations were private limited admission shelters that avoided overcrowding and killing for space by controlling admission. As the movement gained steam in the 2000s, public open admission shelters—organizations legally required to accept all relinquished, stray, or abandoned animals within their jurisdiction—increasingly began to adopt no-kill methods. By the mid to late 2010s, achieving no-kill status, widely operationalized as maintaining a 90% or greater live release rate (21), had become the de facto goal for nearly all shelters, if often unrealized. To this end, urban municipal shelters now commonly include programs for adoption, fostering, rehabilitation, and low-cost sterilization. These same animal services and practices, however, are limited or non-existent in many rural and small-town settings.

To better understand rural animal welfare services, this study explores the organizational structure and challenges of shelters serving rural areas using data from ethnographic interviews and observations focused primarily on rural Oklahoma. Findings reveal large “shelter deserts” and services gaps within rural areas that leave residents with little support from local governments to help solve animal problems. Private citizens and non-profit organizations step in to fill this gap and, in some instances, have succeeded in making their rural communities “no kill.” We introduce three rural rescuers and their communities to illustrate the different forms and pathways through which rural communities have achieved “no kill.” Despite the successes, large barriers remain to improving and sustaining rural animal welfare services, including challenges related to funding, labor and staffing, and market access. We conclude by exploring avenues for future research and prospects for addressing these challenges.

## 2 Materials and methods

### 2.1 Study background and setting

The data for this study comes from a larger project focused on relocation networks that move dogs and cats at risk of shelter death out of state for adoption (22). As a result of technological advances, shifting norms around companion animal treatment, and the animal welfare movement, the number of animals dying in US shelters has fallen drastically over recent decades (23, 24). However, this progress has been geographically uneven, and some areas continue to struggle with large populations of unhoused companion animals and overcrowded shelters. Animal transport moves animals out of these areas, primarily located in the South, to places with excess shelter capacity and higher demand for adoptable animals. Many Oklahoma shelters and rescue organizations, both urban and rural, routinely send animals (mostly dogs) out of state to avoid shelter death. The Oklahoma Animal Transport Study sought to better understand animal relocation as well as the broader animal welfare problems underlying this practice.

Oklahoma, a state in the South-Central United States, has a large and diverse rural population. According to the Census Bureau (70), about 36% of the Oklahoma population (1.4 million people) live in rural places, defined as areas outside of “urbanized areas” (“continuously built-up” places with a population of 50,000 or more) or “urban clusters” (areas with between 2,500 and 50,000 people) (25). At the county level, the Office of Management and Budget classifies 78% (*n* = 60) of Oklahoma's 77 counties as non-metropolitan (26), where metro is defined as counties with a core population of at least 50,000 people. Most residents cluster in the state's three largest metropolitan areas centered around Oklahoma City (pop. 1.5 million), Tulsa (pop. 1.06 million), and Lawton (pop. 126,687). As a poor state, Oklahoma struggles with high levels of poverty and other indicators of social vulnerability. These problems are particularly acute outside of the major population centers, where the rural population is on average poorer, older, sicker, and less educated than their urban counterparts (27, 72) Rural residents also have more restricted access to basic amenities, including broadband internet, public transportation, healthcare services, and grocery stores (28–30).

Rural economic decline and outmigration—resulting from a combination of deregulation, agricultural consolidation, federal trade policy (NAFTA), and reduced state and federal allocations (31)—limit the capacity of small local governments to provide public services (32). As a result, rural governments report higher levels of fiscal strain, lower administrative capacity, and fewer public services. In their study of county governments, sociologists Lobao and Kelly (5, 33) find that metropolitan counties have the highest level of governmental capacity and offer correspondingly more public services, followed by rural counties adjacent to metro areas. Remote rural counties come in last, with the least capacity and fewest public services. Qualitative studies likewise find distinct rural challenges in public service provisioning. In a telling example from a study of rural emergency management in Oklahoma, the authors find that “even the most fundamental emergency needs cannot be met,” with one rural police chief noting that “as a town, with our tax revenue, we can't afford to operate an ambulance” (34). After a tornado hit their town, another law enforcement officer explained that “the bottom line is we don't have the finances or the assistance to get the necessary warning devices.” These conditions also affect the delivery of rural animal services.

### 2.2 Data collection

Interview participants for this study were recruited through key informants, participant referrals, and direct outreach to animal welfare organizations between 2022 and 2025. Kay Stout, a local animal advocate and our primary community partner on the project, compiled the initial contact list and reached out to potential participants to vouch for the study before the research team made contact. Contacts were invited to participate in the study via email, followed by phone and social media outreach when email failed to get a response. At the end of each interview, we asked participants to recommend other rescuers who they thought we should talk to. Recruitment initially focused on rescuers active in animal transport. As more questions emerged over the course of the interviews, the target population expanded to gain a more comprehensive understanding of animal sheltering and welfare issues. During data collection, we regularly evaluated the sample for organizational, demographic, and geographic representation and targeted recruitment to fill any identified gaps.

Interviews took place in 2022–23 and were conducted over the phone or Zoom following a semi-structured question guide. Questions explored organizational mission, programs, and challenges, including issues specifically related to rurality. Interviews were audio recorded and transcribed for analysis. Data collection ended once key groups were well represented in the sample, few new themes were emerging during interviews, and responses to our recruitment efforts had stalled.

In total, we interviewed 66 people involved with animal sheltering and transport, primarily within the state of Oklahoma (73%, *n* = 48; Table 1). Participants worked or volunteered for a variety of organizational types, including municipal and non-profit shelters, rescues, and state or national policy institutes. About 40% (*n* = 26) of participants worked at a rural organization [defined as codes 4–10 on the USDA's Rural-Urban Commuting Area (RUCA) codes].

**Table 1:** Participant demographics and organizational information (*N* = 66).

**Category**	**Count**	**Percent**
**Gender**		
Women	56	85%
Men	10	15%
**Race/ethnicity**		
Native American (alone or in combination)	5	8%
Asian American	1	2%
Latina/o or Hispanic	1	2%
White alone	56	85%
Multiple	2	3%
Missing	1	2%
**Age**		
18–34	13	20%
34–44	14	21%
45–54	13	20%
55–64	18	27%
65–74	5	8%
75+	1	2%
Missing	2	3%
**Location**		
Oklahoma	48	73%
Out of state	18	27%
**Geography** ^a^		
Rural	26	39%
Urban	40	61%
**Organizational type** ^bc^		
Non-profit shelter	22	33%
Municipal shelter	17	25%
Rescue	13	19%
Transporter	7	10%
Policy institute/funders	8	12%

^a^Categorization based on the US Department of Agriculture rural-urban commuting areas (RUCA). Rural encompasses RUCA codes 4–10 (71). ^b^Shelter refers to organizations with a physical facility for housing animals, while rescues do not shelter animals directly (for example, foster-based organizations). Only one shelter in our sample was a private non-profit with a government contract. ^c^*N* = 67 because of one participant with multiple organizational affiliations.

Demographically, most participants identified as women (85%) and white (85%), matching what we know about rescuers as a group more broadly (35, 36). They ranged in age from 27 to 82 years (average 49 years old).

To supplement the interviews, we conducted 12 ethnographic observations of shelters and rescue activities between 2022 and 2025, eight (67%) in Oklahoma and four (33%) out of state. Observations sought to document shelter and transport activities across a range of organizational types and geographies. Direct invitations from interview participants and recommendations from community partners also informed site selection. Most of the observations (75%) were with organizations who had previously participated in an interview, allowing us to compare themes within the interviews to actions on the ground. Activities observed include transport activities (32%), spay and neuter clinics (8%), and other routine shelter activities (58%). Five (42%) of the observations occurred at rural shelters. Observations were documented through written or audio recorded and transcribed field notes completed shortly following visits.

### 2.3 Analytic strategy

Interview transcripts and observation field notes were analyzed in NVivo 15 following a flexible coding approach (37). Based on the structure of the question guide, we developed a codebook outlining the major thematic topics present in the data. Themes include pathways into rescue work, job responsibilities, community problems, and geographic differences in animal welfare challenges. A second round of more focused coding analyzed these themes in more detail. In this paper, we focus specifically on rural themes drawn primarily from the codes on rural-urban geographic differences and community challenges (Table 2).

**Table 2:** Coding instrument for exploring rural animal welfare problems.

**Code**	**Definition**
Political and economic	Political and economic problems in rural communities that contribute to animal welfare problems.
Legal	Differences in laws, regulation, and enforcement that contribute to rural animal welfare problems.
Geographic	Geographic features of rural places that contribute to animal welfare problems (e.g., distance from urban centers).
Animal services infrastructure	Problems related to animal services infrastructure (e.g., lack of local shelters or low-cost spay/neuter services).
Organizational challenges	Internal organizational challenges (e.g., staffing, administrative capacity)

The data was team coded by multiple researchers. To ensure consistency across coders, a second researcher reviewed the content of each code to identify potential issues or discrepancies, which were discussed and resolved at weekly team meetings. Findings were also validated through discussions with community partners throughout the project and a study report shared with all participants, along with an invitation to provide feedback. Throughout the study period, the research team regularly discussed any deviant cases or contradictory findings and used these instances to further develop our explanatory model.

The three case studies explored in Section 3.2 illustrate the diversity of rural animal welfare operations throughout Oklahoma, with cases selected to highlight different organizational perspectives (non-profit and municipal) and activities (transport, spay/neuter, and sheltering). These cases also represent examples of “success” as recognized by other rescuers, gauged in large part by their ability to reach and maintain a 90% or greater live release rate. As such, comparison across the cases reveals analytically generative commonalities but cannot be generalized to rural communities more broadly. For example, high-volume and low-cost spay/neuter services (Section 3.2.2), although viewed as essential by many participants, are relatively uncommon in rural Oklahoma.

Unsurprisingly given the numerous and complex nature of governmental and scholarly definitions (38), most participants hold cultural rather than official understandings of “rural,” with rurality socially constructed through everyday practices and discourse in interaction with formal institutions (39). In this study, we highlight participants' understandings of rurality, which at times may conflict with official definitions. Some urban rescuers, for example, discussed “rural” problem areas within their city limits that might be categorized differently elsewhere. Rurality also exists on a spectrum and encompasses a wide variety of community types. Because of this, we provide information about participants' communities (demographic, economic, geographic) to better contextualize findings and avoid homogenizing rural. This conceptually broad approach to “rural” stems from the necessarily exploratory nature of our study in the context of a dearth of prior scholarship on the topic.

### 2.4 Ethics approval

Study procedures and material were approved by the University of Oklahoma—Norman Campus Institutional Review Board (IRB #13906). All participant and organization names are pseudonyms. Other details have been altered to further protect participant identities.

## 3 Results

### 3.1 Rural “shelter deserts” and service gaps

**Figure 1:**
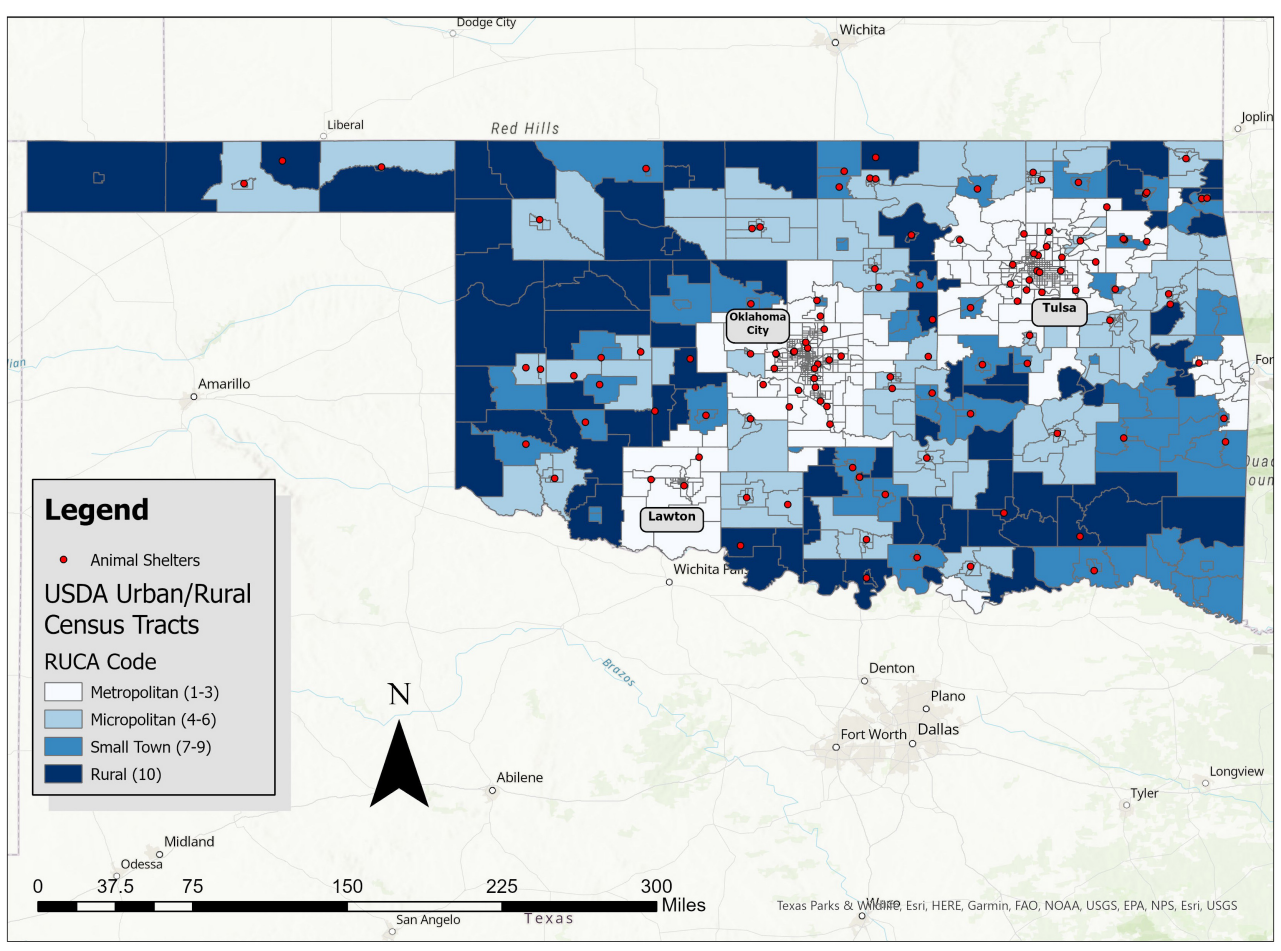
Animal shelter presence across the rural-urban landscape in Oklahoma. Map credit: Andrew Kim.

Throughout rural Oklahoma, large service gaps leave much of the state with no or minimal access to official animal services. As shown in Figure 1, most municipal shelters cluster around Oklahoma City and Tulsa, with fewer locations in the rural west and southeast parts of the state. In fact, nearly a quarter of Oklahoma counties (22/77) have no municipal animal services (40), areas one participant describes as “shelter deserts.” These shelter deserts frequently correspond with areas of high poverty, particularly in the southeastern and southwestern corners of the state (27). Even the rural places with existing shelter services may offer only the bare minimum. A third have no publicly listed contact information or hours of operation (40). As a result, participants describe these areas as among the worst off and most in need when it comes to animal welfare. Willa, for example, lives in a larger city but focuses her rescue efforts on rural areas because those animals “have less of a chance than the ones here in the city.”

Everybody gripes about the [big city] municipal shelters because it is a tough situation there. But those animals at least have a shelter to go to. Out in [surrounding small towns] they don't have shelters. They may have an ACO [animal control officer], but they don't have a shelter. And then when you get [outside the towns and cities] it becomes a county issue. There are no county shelters.

The limited or non-existent nature of official rural animal services means fewer resources for rural residents and their companion animals.

The organization of official animal control and welfare varies across the US but tends to be highly decentralized and with little oversight. Most municipalities are tasked with providing “animal control” services to enforce local and state animal law, leading to the establishment of public sheltering facilities. Because public animal services depend on local taxes for funding, it follows that local economic conditions will influence the level and quality of available services. Although municipal shelters must generally accept all stray or abandoned dogs within their jurisdiction regardless of age, temperament, or health, they may not be required to intake cats and other domestic or wild animals. Larger facilities often voluntarily expand to serve other species, especially cats, but this is less common for rural and small-town shelters. Amanda, outreach director at a national non-profit, describes a common scenario in rural areas, where the shelter amounts to “a couple of dog kennels attached to the police department.” With no housing for cats and only a small number of dog kennels, the official animal services in many rural areas tend to focus on only the most severe “problem” animals.

The physical facilities of many rural shelters reflect the low levels of public investment and often fail to live up to modern standards. As Becky, a shelter volunteer, describes, “Some of these shelters these towns have are just God awful. They're a cement block with no electricity. It's just awful.” Similarly, the “rural pound” in Tara's community, a small-town of less than 1,000 residents, amounts to a lean-to shed in a field outside of town. “There's no electricity, so there's no heat, there's no cooling. There's no anything,” she says. Participants describe substandard operations from many rural and small-town shelters, resulting in a high likelihood of death for animals entering their care. When Tara volunteered to look after the shelter in her town while the ACO was on leave, she was appalled by the poor quality of animal care.

The poop was being washed out behind [the shed], which I knew immediately as a farmer's wife that wasn't cool…There was ants, there was flies. It was sad. It was pathetic. While there was a shed, there was no dog houses. So [the dogs] would be put in there on concrete, stay there three days. If nobody claimed them within that three days, they were taken out and shot.

Missy encountered a similar situation in her hometown, where she says dogs were housed with kennel mates that would kill each other, left without care during severe weather events, and used for “target practice” when their time was up. In another midsized town, Mandy says ACOs routinely kill animals before holiday weekends because no one is willing to care for them. Multiple participants refer to small-town ACOs as an “ol' boys club” with little interest in providing high-quality animal care, rehoming, or implementing non-lethal means of population control.

Sheltering facilities may be organizationally located within any unit of government but often fall under police departments, public works, or code enforcement (41). Larger towns and cities have dedicated shelter staff and ACOs whose full-time positions revolve around animal care and enforcing animal law. In smaller towns, police officers often assume animal control and welfare responsibilities on top of their other duties and may have limited experience, interest, or training in animal welfare. According to participants, the level and quality of animal care depend almost entirely on who happens to occupy these positions. The patchwork nature of animal law, most of which is dictated by local ordinances, further enables vast differences in animal services based on location.

Currently, no federal laws or regulations exist to govern standards of care in sheltering facilities (42). Some states, including Oklahoma, have set shelter standards, including regulations on kennel size, waste disposal, and veterinary care (43). However, inspecting agencies tend to be underfunded and unable to provide meaningful oversight. In Oklahoma, shelters are not legally obligated to notify the owners of impounded animals or attempt to rehome those who remain unclaimed before destroying them. Unlike most states, Oklahoma does not have a mandatory holding period to allow owners to reclaim impounded animals (44). This means that, barring local ordinances that set limits, shelters may legally kill homeless animals in their possession for any reason (or no reason) and at any time.

State law also dictates permissible ways to “destroy” shelter animals, which may be through a sodium pentobarbital injection, carbon monoxide gas, or “any other method approved by the…State Department of Agriculture” (45). Only licensed veterinarians or veterinary technicians may euthanize animals in private shelters. Public shelters have more leeway. To administer sodium pentobarbital injection, animals control officers must be registered by Oklahoma Bureau of Narcotics and Dangerous Drugs Control, but carbon monoxide administration requires only that the personnel are “thoroughly instructed and be adequately trained.” While the law requires that shelter killings be “humane,” it still permits the use of gas chambers, a method widely considered unacceptable and banned across much of the US (46). As recently as 2016, several rural municipal Oklahoma shelters continued to operate gas chambers (40). Since that time, all shelters nationwide appear to have ceased the practice following public pressure (47). Violations of the euthanasia act by private or public animal shelters constitutes a “nuisance” that “any person may maintain a civil action to enjoin” (45).

The list of approved shelter euthanasia methods does not include gunfire, a practice reported as common by multiple participants. However, because the law specifying acceptable euthanasia methods explicitly exempts municipalities with populations below 10,000, “bullet euthanasia” may not violate the state law in rural areas. News stories as recent as 2022 suggest that shooting shelter animals continues in some rural municipalities (48), although it is unclear how widespread this practice may be.

Because Oklahoma shelters are organized at the municipal rather than county level, residents in unincorporated areas have even less access to animal services. Municipal shelters generally do not accept animals from outside their jurisdiction, including rural areas just over the city line. Although state law empowers county governments to “regulate or prohibit the running at large of dogs” by establishing shelters and levying taxes for that purpose, the power only applies to counties with populations of 200,000 or more. In other words, 97% of Oklahoma counties (75/77) have no legal means of developing public shelters to serve residents living outside towns and cities. Multiple participants pointed to this law as a hinderance to developing more robust shelter infrastructure in rural areas.

In rural “shelter deserts” without access to municipal animal services, rescuers saw the official “solution” as only worsening the problem. Cynthia, founder of a rural foster-based rescue, recounts a common response when rural residents call the county sheriff's office for help:

I quit [telling people to call the sheriff's office for help] a long time ago because their standard response—and I called in and heard it myself—is, “Well, you know, you have every right to shoot the animal if it's on your property”…And instead of advising them or giving them resources, they tell them to shoot it… That's how it works in [this] county.

For residents unwilling to exercise their right to execute animals that may wander onto their property, and unable to access other support, the situation can quickly spiral out of control. Ashley works at a large urban non-profit shelter but says that “[E]very single hoarding situation we've done…is in some rural community somewhere” that doesn't have a municipal shelter and may be “an hour or two hours away from a facility like ours that would do spay neuter clinics.” In these cases, a few unaltered animals can quickly reproduce and “within months they've got 20, 30 animals and they never had any option for them.” The lack of animal services thus directly contributes to problems of unwanted, unhoused, and “hoarded” animals.

According to rural rescuers, stray and abandoned animals without shelter access face poor prospects. While unhoused animals in cities may be more likely to die in overcrowded shelters, Willa explains that rural animals experience a “different kind of death.” But, she says, “it is a *certainty* they will die.”

It's not a certainty at the [city] shelters because rescues are working their butts off to pull dogs out so they'll euthanize less dogs. Rural dogs have less of [this help]. They will absolutely die. The rural dogs will die. They will get heartworm. They will get hit by a car. The wildlife [will get them] or they'll get shot.

Rescuers see this problem compounded by the tendency of people to “dump” or abandon dogs and cats in the countryside, often because of misguided beliefs that animals stand a better chance in more “natural” areas. In fact, multiple rescuers first began helping animals because they lived in rural places where people abandoned dogs and cats. Yet, as Willa and other participants explain, the paucity of animal welfare resources in these areas lowers the odds of successfully rehoming animals.

While service gaps define much of the rural animal welfare landscape, some communities have found ways to improve and expand on local animal services or otherwise fill the gaps in official animal services.

### 3.2 Bringing “no kill” to rural Oklahoma

In the rural shelters offering higher standards of care and services beyond caging and killing, operations typically transform after a resident discovers the poor conditions in their local shelter and takes it upon themselves to improve matters. As the prevailing benchmark for “no kill” within animal sheltering, rescuers closely track the live release rate of their shelter and seek to maintain 90% or greater. Yet as a broader philosophy and contested concept, “no kill” holds different meanings across participants and many recognize the shortcomings of a singular, quantified measure of animal care [for a discussion of the tensions and controversy of “no kill,” see (49)]. Beyond this benchmark, “no kill” also refers to a suite of organizational policies and practices that enable shelters to reduce their killing (e.g., mass spay and neuter, adoption, fostering, pet retention). Reflecting these different conceptions and approaches, as well as local conditions, the arrangement and pathway to “no kill” varied across communities represented in our study. Often, rescuers established non-profit organizations to help market and adopt shelter animals or provide other services to communities. In other places, rescuers took over municipal animal services, either voluntarily or as paid staff, and reformed operations from within. Ultimately, all participants sought to cease shelter killing as a means of population control, reserving euthanasia for only the most dangerous and medically or behaviorally untreatable animals, and advocated for broader transformations in the relationship between humans and companion animals. This section introduces three participants and their communities as examples of how lifesaving these transformations have occurred across rural Oklahoma.

#### 3.2.1 The transport lifeline

Sheila, a white woman in her mid-fifties, works out of a rural county in eastern Oklahoma. The population of the entire county barely reaches 50,000 and the largest town, Tilden, has less than 9,000 people. Although majority white, the county is home to a sizable Native American community and growing Latinx community. Service and government make up the largest industries, followed by agriculture. Despite low levels of unemployment, per capita income in the county is just $24,000, compared to $43,000 nationally and $34,000 in Oklahoma. At 16%, the percentage of families living in poverty is nearly double the national average. More than 20% of households rely on SNAP benefits (formerly food stamps) to eat.

Sheila first got into rescue as a volunteer for a local non-profit. When the founders of that group couldn't do it anymore and quit in 2015, Sheila and a fellow volunteer co-founded Halfway Hounds Animal Rescue. The pair spent the first few years getting their bearings in the world of rescue and learning as much as they could from more experienced rescuers. Initially, Halfway Hounds focused on the Tilden Animal Shelter, pulling unclaimed or surrendered dogs and cats and placing them in foster homes for adoption.

But soon, Sheila says, they “had more animals in foster homes and in our homes than we could get adopted.” That's when the rescue started transporting animals up North for adoption. Halfway Hounds first sent only a few animals at a time with other groups but as they established their own relationships with northern partners, they began to coordinate their own trips and eventually purchased two transport vans. Every year their capacity grew. To streamline the transport process, Halfway Hounds built a large transport facility that could house up to 60 dogs and 20 cats while awaiting relocation. They began helping more municipal shelters in the county. At the time of our interview, Halfway Hounds was pulling from nine local pounds and working with police departments in several other communities without shelters. Sheila says that “none of [these communities] would be no kill if it wasn't for our rescue. Because we're the only ones that go in and pull the animals out… If we don't pull them, then they would be euthanized.”

Through grants and donations, Halfway Hounds provides animal services that larger communities expect from their city shelters. When the ACO of a local municipal shelter finds a stray, Halfway Hounds posts the animal on their website to notify owners. After they pull an animal from the shelter, Halfway Hounds does all the routine vetting: “We do heartworm tests on the animals. We do parvo and distemper vaccinations. We do intranasal Bordetella. We give them flea and tick prevention. And we give them a dewormer.” Larger shelters vaccinate all animals on intake to avoid disease outbreak, but this is infeasible for many small and underfunded municipal shelters.

In 2021, Halfway Hounds took in more than 2,100 animals, including 1,600 dogs and 500 cats. Ninety percent of the cats found local homes through adoption and the other 10% headed out of state on transport. For dogs, the statistics are reversed. Ninety percent made the trip north to find new homes in states like Colorado, Illinois, and Connecticut and only 10% found in-state adopters. “Transport saves more lives than the adoptions that we could do locally,” Sheila says. Halfway Hounds sends animals out every 2 weeks like clockwork. Sometimes the destination shelter will meet them halfway. Usually, two Halfway Hounds volunteers make the 10- to 12-h drive themselves, hauling anywhere between 20 and 40 animal passengers. Halfway Hounds has more success placing cats in state but even those rarely stay in county. Instead, they go to a nearby city to be displayed in pet stores where unsuspecting shoppers might see them and fall in love. Without these lifelines to move shelter animals out of the county or out of the state, most shelter animals would still be dying in local pounds.

#### 3.2.2 Population control through spay/neuter

With a population of 20,000 people, Missy's county is even smaller than Sheilia's. The largest town, where Missy's shelter is located, encompasses fewer than 3,000 residents. Otherwise, the demographics and economy look very similar to where Sheila lives. Before she got into rescue full-time, Missy worked on the family farm with her husband and three kids. She happened upon the municipal shelter 1 day while making a run to the dump. She had never noticed the small concrete building before and began asking questions. What she learned horrified her. She asked the city if she could take it over and soon raised enough funds to build new facility.

Like Halfway Hounds, Merry Mutts relies heavily on transport. They only accept dogs and no longer place any animals locally, opting instead to send them all to their partnering shelter in the Midwest. In fact, Missy will only admit animals that she knows she can send north. She's taken home too many “feral” dogs already, animals too under-socialized to rehome, and the shelter permanently houses others who have proven unadoptable. Missy says that at Merry Mutts, “we don't euthanize for space or because they're feral or anything like that. I would never do that. I couldn't.” She just doesn't have the heart. But she understands those who do. There are simply too many animals and not enough homes in Oklahoma.

Although Merry Mutts operates as the local municipal shelter and works with the police department to house city animals, they are a non-profit organization and do not receive any funding from the city. With money from a few large donors, they were also able to start a low-cost, high volume spay/neuter clinic. Missy is most proud of the work the clinic has done.

For the past decade, Missy and a small team of volunteers have assembled every Wednesday to sterilize the dogs and cats of Oklahoma. Pet owners travel from miles around to reach the clinic. As the only low-cost option in their corner of the state, Merry Mutts performs surgeries at a fraction of the price of private clinics. If someone can't afford the fees, Merry Mutts will further reduce the cost. The vet team also must travel to reach the clinic. Mel, a veterinary doctor in his mid-60s drives 2 h each way to provide services, at times only getting home well after midnight. After working together for a decade, the Merry Mutts spay/neuter team operates as a well-oiled machine. On an average day, the team completes more than 70 surgeries. Veterinarians at private practices, they tell us, usually cap out at 3–4 surgeries per day.

Even sterilizing more than 30,000 pets since opening, Missy sees no end in sight. The pace of the work has not slowed a beat. Every week, the appointments fill up. Every week, the shelter must turn away dogs in need that cannot be transported north. The area needs more low-cost spay/neuter clinics and greater outreach and education to reach rural pet owners, Missy says. They need more veterinarians like Mel who have mastered the necessary skills for high-volume surgeries, something not taught during their training. And they need more hardworking people like Missy advocating for animals and providing these essential services.

#### 3.2.3 Shelter reform

Raylee wasn't the first to attempt reform at the municipal shelter in Chikaskia, Oklahoma. The community liked the idea of “no kill” and built a new facility about 5 years before Raylee took over as director of the Chikaskia Animal Shelter. Unfortunately, Raylee says, no one really knew what “no kill” meant. Previously, like many small town and rural shelters, the Chikaskia shelter killed all unclaimed animals after a three-day stray hold. The shelter kept few records at that time, but Raylee estimates they were killing around 90% of their intake. When the town decided to become “no kill” in 2014, the shelter simply stopped killing animals for any reason, including aggression and other serious behavioral problems. This quickly led to deteriorating relations with the community. Raylee's first move as director was to clean house. About half of the kennels were housing long-stay dogs that had become too dangerous to adopt and could no longer be handled safely. Even so, the staff balked when Raylee directed them to euthanize 50% of the dogs. “Just trust me,” she told them, “Trust the process.”

Chikaskia, a town of about 12,000 and headquarters to one of Oklahoma's 38 tribal nations, sits on the historic Route 66 in western Oklahoma. Large-scale manufacturing once powered the economy and, despite some decline, remains an important industry. Outside government jobs, tourism, driven by a smattering of museums and casinos, makes up the largest source of employment. But despite a recently revitalized downtown and numerous tourist attractions, Chikaskia continues to struggle. At 23%, the poverty rate is high, even for rural Oklahoma, and only 16% of adults have earned a bachelor's degree or higher, a full 40% lower than the state average. Nonetheless, Chikaskia exudes a sense of pride in its heritage and optimism for the future.

Under Raylee's leadership, the Chikaskia Animal Shelter instituted a series of reforms that have slowly improved relations with the community. The shelter began euthanizing again, but only for severe behavioral and medical cases. While they are unable to provide low-cost spay and neuter to the public given budget constraints, the shelter works closely with All About Animals, a small rescue in town that hosts free clinics monthly. For several years, All About Animals also transported large numbers of Chikaskia shelter animals out of state, over 800 dogs annually. At that time, most dogs spent less than a month in the shelter. This all changed with the pandemic. When COVID-19 hit, everything shut down, including transport. And now, the destination organizations that previously accepted Chikaskia dogs have their own problems to deal with. Raylee says that the shelter “still occasionally sends animals but nothing near what we were sending pre-pandemic. They mostly take in breeder surrender animals now. But even those don't move as fast. Nobody is adopting right now.” Local donations to animal welfare causes have also withered as families struggle to make ends meet amid post-pandemic inflation. As a result, dogs now stay at Chikaskia for closer to 4 months. Fortunately, cats have increased in popularity and, between local adoptions and their trap-neuter/spay-return (TNR) program, do not currently pose a problem for the shelter or community.

With dog transport and local adoptions both slowing to a trickle, the shelter faced a choice: they could either begin killing again or limit intake. Raylee and her staff are unwilling to kill healthy animals for space and instead opted to implement a triage system known as “managed intake” to regulate the flow of animals coming into the shelter: urgent cases get priority, while all other animals go on a waitlist until a kennel opens up, something that may take months (50). Managed intake, a controversial practice that shelters nationwide began adopting during the pandemic, has allowed the shelter to remain “no kill” but leaves many community animals with nowhere to go. According to shelter staff, the community has enough animals in need to refill the kennels weekly: “We could get a whole new set of animals every week. But there aren't the resources or homes for that.” For Raylee, this speaks to deeper problems in modern sheltering and animal welfare. “There's a misconception about what the shelter can do,” she says. “The shelter isn't a fix-all. People find an animal and call us and it's out of sight out of mind.” Instead, Raylee would like to see more community-based solutions that bypass the shelter entirely. Making this a reality may feel like a pipedream but she's determined to move Chikaskia in that direction.

### 3.3 Rural sheltering challenges

The stories of Sheila, Missy, and Raylee illustrate three rural communities that successfully transitioned to “no kill” and were able to at least partially address the animal service gaps. However, these stories also highlight the challenges of rural rescue and sheltering that remain even in instances of “success.” Our analysis identifies three key challenges related to rural sheltering. First, the funding structure of both public and private shelters favors urban organizations and leaves rural shelters financially strapped. Second, rural shelters rely heavily on a small number of workers, often unpaid volunteers or poorly paid staff with low levels of professionalization. Third, rural shelters often struggle to access adopters and veterinary services.

#### 3.3.1 Funding

Participants from both public and private rural shelters cite funding as among their greatest challenges. Multiple participants point out that virtually all shelters lose money and will never be financially self-sustaining. Adoption fees at some organizations, primarily private non-profits in locations with shortages of adoptable animals, can range from hundreds to thousands of dollars, offering a significant revenue stream. In Oklahoma and other places with large overpopulation and shelter overcrowding problems, however, adoption fees tend to be much lower. Most public shelters in Oklahoma charge less than $100 per animal, with additional specials and discounts on long-stay and older animals, or when the shelter has reached capacity. Sometimes shelters waive the fees entirely, giving away animals for free. Adoption fees cover only a fraction of the cost to house, feed, vet, and sterilize an animal. Private shelters often charge higher adoption fees, but these are still rarely enough to recoup the full cost of care, much less cover operating expenses.

Municipal shelters rely on local tax revenue to operate, creating variability based on the relative wealth of communities, political context, and local decision-making. As poorer areas, rural and small-town municipalities generally have fewer resources to devote to public services, and animal services must compete with other priorities. In the words of Raylee at Chikaskia Animal Shelter, the shelter must “fight for every penny” during budgeting season and making a compelling case for funding is difficult. Others, like Missy, have given up entirely on interacting with the city. For her, the small amount of public funding they might receive just isn't worth the frustration. Nathan, who has worked extensively with rural shelters as a consultant and non-profit director, likewise sees animal services as falling low on the priority list for most rural municipalities. In rural regions that have been “chronically neglected by every social service,” animal welfare is “the most neglected social service,” he says.

The under-resourcing of animal welfare combined with high need and large service areas strains the capacity of rural shelters. Some rural municipal shelters report an inability to provide even basic animal care and services. Freda, who runs a foster based rescue that pulls dogs from rural Oklahoma shelters, works with shelters that cannot even afford food for their animals:

It's shameful that a town has a shelter, which they collect tax dollars, and yet they won't buy food. So, they have to look for food donations to feed the dogs....But I get it. Right now things aren't great economically.

Other participants report funding as a barrier to providing vaccines and medical care. Mackenzie, the director of a small-town municipal shelter, points to problems “just trying to keep food and medical supplies that we need here to keep animals wormed or if they come in with wounds, wound spray and all that.” She goes on to say, “We're getting away with the absolute bare minimum of vaccines. If animals needed to go to the vet, we had to judge it on can we home remedy it or do they actually need to go to the vet?” This also meant that at one time, the shelter was “adopting out unaltered animals because our city just couldn't afford [the spay/neuter surgeries].” Shelters like Mackenzie's often have fewer transport opportunities because they cannot meet the requirements of out-of-state partners that animals be vaccinated and altered prior to transport. To make up for funding shortfalls, municipal shelters turn to donations and help from local non-profits. The heavy reliance on private organizations and donations to provide animals services have led Nathan to conclude, “We are essentially a privately funded public service in a lot of places.”

Non-profit shelters derive their operating budgets from a combination of grants, small donations, and philanthropic foundations. Major sources of funding include the charitable arms of large pet retailers (PetSmart Charities and Petco Love) and national animal welfare organizations [e.g., ASPCA, Humane World for Animals (formerly the Humane Society of US), Maddie's Fund, Best Friends]. Although local shelters often operate under “humane society” or “SPCA” labels, they generally have no formal relationship with the national organizations that share similar names. Money donated to these national groups only supports local organization through grants. Participants describe grants as an important but flawed funding mechanism. Dee directs a rural non-profit shelter and explains how the short-term and program specific nature of grants makes them “very, very helpful but challenging.”

The big funders like PetSmart, the ASPCA, etc., they frequently will focus on one area of animal rescue for three years, and then they'll go on to another area. Well, if you lose it, if you're relying on that funding for those three years and you still need those funds for the next two or three years, where do you get the money?

The outcome of losing grant funding is that the lifesaving programs “shut down and, yes, the animals are put down.” Other challenges with grants include the need for administrative capacity, which small shelters and rescue may not have, and the fact that grants seldom cover labor, infrastructure, and overhead costs.

Private foundations with broader missions also provide grants and financial support to animal welfare. However, as Shonda, the director of a rural non-profit shelter, points out, charitable foundations frequently change priorities.

One year it was all about saving animals…[and] spay and neuter. Well, now it's about, maybe, Afghan refugees or something like that. And I'm not being negative. I'm just saying, they move in cycles. Dogs are long gone from their thoughts.

Robyn, director of a non-profit in a midsized town (over 35,000 population) that serves a large rural area, learned how few foundations focus on animal welfare when they sought philanthropic funding to build a new facility. The first family foundation they approached, one known for supporting sheltering in the state, declined because the new generation in charge had different priorities. They next asked the wealthy oil and gas corporations headquartered locally, only to hear again and again, “Animals don't fall in our scope of giving. We only give to children and the arts.” In the US, less than 4% of all charitable giving goes to support causes related to animals and the environment (51), making philanthropy an unreliable funding source for many animal welfare organizations.

#### 3.3.2 Labor and staffing

Labor and staffing present a second major challenge for rural shelters. Across much of rural Oklahoma, a relatively small number of rescuers assume responsibility for all animal welfare work in their communities. In the words of Sam, director of a statewide policy organization, there are “pockets where you've got individuals that are doing the lion's share of the work.” These individuals might be paid employees at municipal shelters like Raylee at Chikaskia but are often unpaid volunteers like Sheila, who runs Halfway Hounds. Nathan, the rural consultant, has learned from working with organizations across the nation that this situation is common.

We have rural shelters and we have rural groups that are doing hundreds if not thousands of animals a year, acting as the only service in their area, and they receive no public support or [and don't] have any [paid] staff. And I think that that is crazy how common that is.

These arrangements result in a rural workforce that is less compensated, specialized, and professionalized than their urban counterparts.

Both urban and rural areas rely heavily on (predominantly female) volunteers and low-paid workers to care for animals and deliver services. Nationally, animal care and service workers earn a median salary of less than $34,000 (52), a number likely much lower for rural workers. Cities generally have larger, multi-unit organizations run by professionalized staff, often trained in areas such as business, non-profit management, municipal government, or animal control and welfare. Nearly all rural shelter and rescue organizations in our study, by contrast, operated with only a few core volunteers or workers with little formal training. As a result, titles like “director” and “president” carry very different meanings depending on the organization and context. The director of a large urban shelter may hold an advanced degree and earn six figures, while most study participants who held top-level positions at rural organizations took little or no pay. Mandy, founder and director of a non-profit for a small city in northern Oklahoma, pays herself a salary of only $23,000 after more than 20 years in the position. Now nearing retirement age, she worries that no one will be willing to replace her at such low pay. On the other side of the state, Shonda directs a small-town non-profit as a volunteer while continuing to work a full-time day job. Both Mandy and Shonda say they have difficulties finding workers to fill low-wage positions in their organizations. Shonda describes their staffing issues:

I'm having trouble keeping enough people to work. My dogs don't get all the attention they should get. I have a hard time paying somebody $9 an hour to come out and push poop and water, and I can only afford two hours in the evening. I don't have anybody to work, and I can't do it all [by myself].

Although many small non-profit organizations face these problems regardless of geography, urban animal welfare groups tend to have a larger volunteer base to draw on and can better compensate employees.

Due to their smaller numbers and organizational size, the work of rural rescuers tends to be less specialized than the typical urban shelter. Larger organizations often have positions dedicated to facilitating adoptions, coordinating volunteers, caring for animals, and administrative tasks. At rural shelters, all these responsibilities may fall onto one or two people. Mackenzie describes the variety of tasks she has taken on as a small-town shelter manager in a department of two.

I've done everything. I've been a plumber here. I've been an electrician at one point. We do construction around here trying to get these rooms ready for these animals. We go and deal with feral cats. We remove dead animals. It's literally anything and everything you can possibly think of. We are kennel techs, we are groomers, we are vet techs…. We are the [temporary] owners…[who] teach them love. We are trainers, we are receptionists. We are just absolutely everything. And between the two of us, if it's not done, then it's not getting done.

Similarly, when Chikaskia Animal Shelter is fully staffed, just three employees provide animal services for the entire town.

Low levels of staffing limit what rural shelters can hope to accomplish on a day-to-day basis beyond basic animal care. Janice, an animal control supervisor in a town of about 16,000, says that it's “hard to find placement for any our dogs because people are so focused on Tulsa dogs [and] Oklahoma City dogs,” cities where the shelters bring in “a hundred dogs a day.” As a smaller shelter, Janice is responsible for much fewer animals but their “length of stay is long, and I am the only person that stays here continuously…. Whereas, these other shelters have multiple employees, so they are able to tackle things a little faster.” For Mackenzie, this also translates into greater difficulty transporting animals. Even if the shelter was able to raise the funds and get enough animals together, they are “still not in a position to transport” because, with only two workers, they can't afford to have anyone “missing from the building.”

Rural shelter and rescues can also struggle to access education and resources relevant to their contexts. Nathan sees “a lot of disconnect” between the big national organizations and small rural shelters: “Just trying to find research and just trying to find solutions geared toward rural areas, we don't even have the basics.” The resources from large nationals are “almost always geared with some sort of a professional or staff member being a part of it,” he says, “And we can't assume that.” He offers the example of technological skills and support. As a consultant, Nathan worked with rural municipal shelters without access to an IT department to help with website administration, data collection systems, or even setting up the office Wi-Fi. Yet the resources coming out of the nationals are “built in these gigantic, better resourced, multi-staffed, multi-division places…When you send [a rural shelter manager] a workbook that suggests that he move one of his adoption counselors over to foster counseling, it's not particularly helpful,” Nathan says. As a result, he sees most of the available toolkits and guides as “completely worthless” for rural shelters.

#### 3.3.3 Market access

The nature of rurality creates a third key challenge for sheltering: market access. Relative to their human populations, rural shelters take in a larger number of animals. Scott, a director with a large national organization, works with shelters across the country and observes the high levels of per capita intake within southern rural communities:

It's not uncommon to work with a shelter in a northern area that's very far along in their lifesaving spay/neuter program and they might take in 10 to 12 animals per thousand people...And then it's also not uncommon to go to a place in a mid-sized community in a rural area [in the South] that may end up with a number that's closer to 35, or I've seen as high as 70, animals per thousand people coming into the shelter.

Despite high intake, rural participants experience difficulties accessing “consumer markets” in which to adopt out their animals. The large number of local stray and unhoused animals means that rural residents can easily adopt animals from friends, neighbors, or the streets without ever entering a shelter.

The heavy reliance on transport to rehome rural shelter animals attests to the difficulties they face finding local adopters. Nationally, urban counties rely more heavily on adoption than transport (62.3% vs. 52.3%), whereas rural counties transfer more animals than they adopt out (33.1% vs. 18.5%) (6). Nearly all rural participants in our study described difficulties placing their animals locally. Willa characterizes the local “pool of adopters” as “shallow, shallow, shallow.” Shonda likewise reports low levels of success finding local homes, especially for larger adult dogs, and says, “When we do adopt, it's usually a puppy.” Participants attribute this in part to high levels of economic insecurity compounded by post-pandemic inflation. Through transport, rural shelters gain access to areas with greater demand for adoptable dogs. Clinton, a small-town ACO, says that, if not for transport, their “euthanasia rate would be astronomical.” While their “adoption numbers some months are really good,” other times they experience a “3- or 5-month dry spell where there's not anybody looking for a dog.”

Most transport destinations, in addition to being in wealthier regions with lower levels of shelter intake, are in or near large cities. Relocation thus provides a means for rural southern shelters to access larger “consumer markets” for adoptable animals. Transfers within states follow a similar pattern, moving animals to more densely populated and wealthier areas [e.g., (53)]. Rural organizations often rely on large pet retailers to reach potential adopters, many of which can only be found in larger cities (with the notable exception of Petsense, the specialty pet retailer by Tractor Supply that targets small and mid-sized communities). Sheila, the Halfway Hounds founder, explains the importance of these stores:

PetSmart or Petsense, they have kennels set up for [cats] since they…can get some exercise on a cat tree or in a smaller area than a dog would need. So the cats are easier to display for the public to see than the dogs are… [I]f we have an opening at Petsense or PetSmart, [we] take them in up there and then they get adopted pretty quick.

Trish, director of a rural non-profit shelter in Kansas, contracts with a PetSmart in a city about an hour away to house and adopt cats on site. Indeed, fully half of their total adoptions happen through PetSmart, which is “one of the biggest reasons why we are able to continue to be no-kill…[and] one of the biggest why we're able to bring in animals from other areas.”

If we were to lose our PetSmart contract tomorrow, we would have to go back to [being]a shelter that euthanizes. So it is important to recognize that while I am very proud of everything that my shelter has done, we still have more animals coming into our shelter than we have local outcomes.

Specialty pet retailers rarely house dogs on site but shelters and rescuers frequently hold adoption events in front of pet stores to adopt dogs to shoppers. These opportunities are less available to rural rescuers because of the distance to large pet stores and the labor needed to staff such events.

In addition to having fewer opportunities to market shelter animals locally, rural shelters face higher barriers to veterinary access, affecting their ability to provide low-cost sterilizations or ready animals for adoption. When asked about challenges associated with rural animal welfare, Bonnie, the president of a rescue in a town of around 18,000, immediately cited the veterinarian shortage.

There's a major shortage of veterinarians in Oklahoma and really nationwide. We are having to pull vets from Oklahoma City to come down here to work in our clinic to either do wellness [exams or spay/neuter surgeries]…We have three vets [in our community], three vets that are slammed every single day. For a community our size, that seems crazy to me.

Although the shortage affects all areas of the workforce, both shelter medicine and rural practitioners have been hard hit (54, 55). Veterinarians often leave school with large student debt, which can diminish the appeal and feasibility of practicing in rural areas with lower earning potential. The increasing entry of private equity and corporate consolidation into the sector in recent decades has also been shown to adversely affect local markets, raise costs, and increase veterinary burnout (56, 57).

Rural rescuers often face the choice of paying full price to send their animals to private veterinary practices locally or travel long distances to reach low-cost services. Mandy, director of a non-profit shelter, describes the tradeoff.

We learned that you can't afford to get veterinary care at local veterinary clinics and adopt the animals out. At the time [when we started], I think our adoption fee was $50 and it was costing us about $100 per animal to get their vaccines, spay and neuter. And that's cheap nowadays, you know? So we learned very quickly that using private practice veterinary care is not affordable.

Instead, they travel 80 miles three times a month for the spay/neuter program at Oklahoma State University veterinary school where students perform surgeries at reduced rates. “I had a Toyota Corolla, and I could pack that car with crates like nobody's business,” Mandy recalls. “I mean, it was not uncommon for us to take 10 animals.” The shelter also works with local retired veterinarians to provide low-cost spay/neuter services to the community but will have to find new solutions when those veterinarians age out of the work.

## 4 Discussion

Our findings suggest that rural animal sheltering and welfare face distinct challenges that reflect organizational, community, and geographic differences. Based on interviews and observations with rescuers working in rural Oklahoma settings, we highlight three key takeaways: (1) rural communities depend heavily on relocation programs, (2) rural and urban animal welfare organizations differ in their organizational structures and level of professionalization, and (3) uneven economic development creates geographic disparities in animal service provisioning. While our findings apply primarily to rural Oklahoma, they suggest productive avenues for future research to assess the extent and conditions under which these conclusions may be generalized to other settings.

First, rural shelters and communities depend heavily on relocation programs to alleviate shelter overcrowding and reduce shelter killings. In southern states like Oklahoma, transport moves dogs out of state while cats often go to more proximate urban outlets. In other regions of the country, evidence suggests similar intra-state rural-to-urban flows of shelter animals, with some northern shelters in our study shifting away from accepting animals from southern states as the situation of animals in their own state worsened during and following the COVID-19 pandemic. This has had ripple effects, as southern shelters then found themselves with fewer outlets to which to send their animals. Additional research is needed to better understand how these dynamics operate across other regions and states, particularly in areas with less severe overcrowding and overpopulation problems.

Through transport, some rural communities successfully reach and maintain “no-kill” status, where they no longer kill animals to address overcrowding. Yet even these rural communities often continue to struggle with large animal overpopulation problems. As Raylee's case shows, relocation programs offer a temporary and often precarious solution. When transport slows or becomes unavailable, communities can quickly re-enter crisis mode. Nearly all participants view transport as insufficient on its own, yet participants offered few strategic alternatives for rural communities that can address the root of the problem at the scale and scope required. This raises broader questions about how “no kill” can best be implemented in low-population and resource scarce areas without easy access to larger adoption markets.

Second, the organizational structure and environment of rural shelters and animal welfare groups distinguish them from the urban norm. Predictably, urban areas have denser animal welfare networks and offer more services on average. Our study also suggests that urban organizations tend to be more professionalized. Following classic markers of professionalization (58), sheltering and animal welfare have developed into full-time occupations with professional associations, training programs, and codes of ethics. While rural organizations are not completely divorced from these dynamics, we found that they are significantly less professionalized than the field as a whole. Some directors at rural municipal shelters hold full-time (but low-paid) positions and enter the job with relevant experience or training. Yet these are often the only paid animal welfare positions in the community, and other small-town governments have no dedicated animal welfare staff. Often, rural ACOs and other municipal shelter workers have limited interactions with the profession more broadly. Most rural private non-profits are volunteer-based grassroots organizations with no paid staff. Similar grassroots organizations are also common in cities but in rural communities, these are often the only groups doing this lifesaving work.

Lower levels of professionalization do not necessarily imply less skill or knowledge on the part of rural rescuers but have implications for how animal welfare operates in these settings. Rural animal welfare offers few pathways for financial security, professional development, or career advancement. This limits who can become involved in the work (volunteers must have the time and financial resources to dedicate themselves to uncompensated work). This heavy reliance on volunteer and poorly compensated labor contributes to a gendered culture of sacrifice, where animal care workers, nearly always women, devote long hours to emotionally draining work regardless of the personal toll. Less professionalized rural organizations are also at a disadvantage when it comes to accessing relevant resources and external support. Grant funding structures assume professionalized organizations with the time, background knowledge, and administrative capacity to write proposals, manage large grants, track progress, and write reports. Lacking the time, resources, and professional know-how, rural rescuers may find themselves operating in relatively small bubbles, sometimes isolated from the larger national animal welfare community.

Finally, our study points to the structural forces underlying many rural sheltering and animal welfare problems. The spatial flow of shelter animals reflects broader patterns of uneven development within and between regions that constrain the ability of small and rural municipalities to provide public services (59). As Lobao and Kelly (33) argue in the context of rural public services more broadly, “local governments' hands are tied strongly by external, systemic forces, such as the national economy and the actions of state and federal governments.” In addition to forms of rural disadvantage experienced nationwide, rural Oklahoma must contend with state laws preventing the development of county-level sheltering systems, and a southern political economy that has left the region among the poorest in the nation (60, 61). Within these constraints, local governments decide how to best allocate resources and respond to a variety of community needs (62, 63). Future research should investigate how these pressures and other factors affect animal service provisioning across geographies.

The concept of “shelter desert,” referring to areas lacking or underserved by sheltering and other animal services, offers a useful point of departure for such a research agenda. An extensive body of literature uses the desert metaphor to describe areas lacking or underserved by a variety of institutions and services, such as food deserts (64), medical deserts (65), and banking deserts (66). The desert concept, however, has not yet been widely applied to animal services. Following the broader deserts literature, “shelter deserts” usefully orients scholars to the geography of animal services and encourages greater analyses of the spatial patterns and predictors of shelter access. Larger and more representative quantitative analyses can strengthen our understanding of how different aspects of rurality matter for animal service provisioning and better identify intra-rural variation, enabling more precise conceptualizations of spatial categories. Notably, many of the problems faced by rural rescuers mirror conditions found in low-income urban settings—including high rates of shelter killing, understaffing and underfunding, and poor access to support services like basic veterinary care (9). This points to the need to investigate “shelter deserts” in both their rural and urban variants.

## 5 Policy recommendations

Based on our findings, we offer several recommendations for funders and policy advocates to support rural animal sheltering and welfare. *First, develop tools and strategic approaches specifically for rural contexts*. When it comes to sheltering, leading animal welfare organizations focus most of their attention on improving organizational practices. Common topics within the weekly newsletter of the Association for Animal Welfare Advancement, for example, include tips for recruiting and retaining fosters, marketing animals, creating an inclusive workplace, and developing leadership skills. These efforts, while important, apply primarily to urban organizations with large staff to manage and a need to draw volunteers and adopters from a dispersed and largely anonymous public. Different approaches may be needed for small rural shelters with few staff and a small pool of potential volunteers and adopters, most of whom are well known to one another. This latter factor, the intimacy of small-town life, contains challenges but also presents opportunities for a community to come together to create services that are vital to their safety and wellbeing.

*Second, advocate for increased public funding and innovative policies to support animal services*. One model for increasing and more equitably distributing public funding for animal welfare services comes from New York state's Companion Animal Capital Fund. This program has provided over $38 million to support shelter improvements since 2017 (67), enabling shelters to upgrade their facilities, purchase equipment, and pay staff higher wages. The recent shift to understanding animal welfare within the context of human wellbeing, which focuses on increased integration of human and animal support services (13, 68, 69), offers another possibility for elevating animal welfare within government structures. Many local food banks, for example, now offer pet food to low-income residents, reducing the likelihood of surrenders. Expanding such programs to government food assistance programs, such as the federal SNAP program, could ease the pressure on local shelters.

*Third, expand private funding streams and long-term grant support for pet owners in socially and economically disadvantaged communities*. While public funding is crucial, increased philanthropic funding for programs like Pets for Life, a program from Humane World for Animals that supports pet ownership in racially and economically disadvantaged communities, can bolster animal welfare in rural places such as Oklahoma. Providing longer funding periods can help build the local networks and infrastructure required to sustainably embed new animal services in communities. Additionally, funders can assist grantees with obtaining other funding streams and developing transition plans well before the program ends, a serious challenge for small rural organizations that may lack the know how or administrative capacity.

Across the country, but especially in rural areas, animal services are under-resourced and under-appreciated, despite being essential to both human and animal welfare. Addressing animal welfare across geographies requires acknowledgment of the vital role it plays in maintaining safe and humane communities, as well as sustained investment to ensure equitable and lasting solutions.

## Data Availability

The datasets presented in this article are not readily available because qualitative ethnographic data contains identifiable information on participants. Requests to access the datasets should be directed to laura.bray@ou.edu.

## Footnotes

1 Best Friends' analyses (8, 21) adopt the US Census Bureau's rural/urban classification based on population density.

2 Our academic literature searches have yet to locate any peer review article with a primary focus on rural animal sheltering or welfare. The most recent search was conducted in February 2026 using two academic databases, Google Scholar and Web of Science. Boolean search strings included “rural animal shelter^*^” OR “rural animal welfare”; (“animal shelter” OR “animal welfare”) AND “rural community” AND “companion animal”.
